# Special Hospitals

**Published:** 1892-07-09

**Authors:** 


### HOSPITAL OPINION.

[Contributions critical, suggestive, controversial, and technical are invited for this column, which is open to everyone who has anything to say that is of practical value and interest.]

### SPECIAL HOSPITALS.

MR. KERSHAW writes from the Central Throat and Ear Hospital: The abstract from the Report of the Lords Committee on the Metropolitan Hospitals will have been read with great interest, and by none more so than those who, like myself, have an official connection with hospital work. As to the question of "hostility so widely shown by the medical profession to special hospitals," permit me to state a few facts which tend to prove that their dislike is not so real as would appear. Mr. Lennox Browne, who gave evidence before the Lords Committee, handed in a table—a copy of which I enclose—showing that 153 physicians and surgeons attached to general hospitals also hold appointments at fourteen varieties of special hospitals. But this table is by no means exhaustive, for some members of the staff of general hospitals do not publish in the "Medical Directory" their special hospital appointments, and many lecturers and demonstrators at general hospital medical schools hold also special hospital appointments, which were not included in the calculations.

Speaking of the special hospital, to which I am myself attached, I find that in the past year no less than 1,000 patients out of a total of 6,674 were sent for advice and treatment by members of the "medical profession," the majority of the applicants bringing either a letter or card of introduction to one or other member of our medical staff. Hardly a day passes that I do not communicate with some member of the "medical profession" as to the well-being of a patient under treatment, and the medical staff also receive and dispatch similar communications quite as frequently. So much for what we do for their patients.

And now for our position as a teaching institution. In the last ten years 1,934 members of the "medical profession" have entered their names in our visitors' book as witnessing the practice of the hospital, and of this number 671 have attended the various lectures delivered at the hospital by our staff, while 15 clinical assistants, being duly qualified medical men, are annually elected for appointments for six months each. Such recognition of our work by the "medical profession" is very gratifying to us, but, so far as this hospital is concerned, the word "hostility" should be changed into "obligation." And it is hardly likely that our experience is exceptional.

It is a matter of regret that a more definite description was not given in the report of those small special hospitals which the Committee do not consider of any real benefit either to the sick or to science—and the more so since particular attention is drawn to one Royal Hospital in which the Committee consider reforms are needed, and that hospital is designated both by its full title and location.

As matters now stand the public have no guide to distinguish between the useful and the useless.

